# Peritoneal tuberculosis presenting with portal vein thrombosis and transudative Ascites - a diagnostic dilemma: case report

**DOI:** 10.1186/s12879-015-1122-6

**Published:** 2015-09-30

**Authors:** Ushani Mayurika Wariyapperuma, Champa Indrani Welikala Jayasundera

**Affiliations:** Sri Jayawardenapura General Hospital, No 564/8, Thaldiyawala Road, Rukmalgama, Athurugiriya Sri Lanka

**Keywords:** Peritoneal tuberculosis, Portal vein thrombosis, Transudative ascites

## Abstract

**Background:**

Peritoneal tuberculosis is an important problem in regions of the world where tuberculosis is still prevalent (Chest 1991; 99:1134). Atypical presentations such as portal vein thrombosis can delay diagnosis or result in misdiagnosis (Gut 1990; 31:1130, Acta ClinBelg 2012; 67(2):137–9, J Cytol Histol 2014; 5:278, Digestive Diseases and Sciences 1991; 36(1):112–115). A high index of suspicion is required for the diagnosis of peritoneal tuberculosis, as the analysis of peritoneal fluid for tuberculous bacillus is often ineffective, and may increase mortality due to delayed diagnosis. (Clin Effect Dis 2002;35: 409-13) In light of new evidence, peritoneal biopsy through laparoscopy or laparotomy has emerged as the gold standard for diagnosis (Clin Effect Dis 2002; 35: 409-13).

**Case presentation:**

We report a case of a 35 year old Sri Lankan female employed in a Middle - Eastern country who presented with progressive abdominal distention and constitutional symptoms for four months duration. She had been investigated abroad and diagnosed with ascites with chronic portal vein thrombosis following which warfarin therapy had been commenced suspecting an underlying thrombophilia. Despite treatment her symptoms had worsened. Therefore she had decided to return to Sri Lanka for further evaluation. After ruling out inherited thrombophilic states and the antiphospholipid syndrome, further investigations revealed a transudative ascites and high inflammatory markers. The tuberculosis work up on peritoneal fluid was negative. Therefore, we proceeded with laparoscopy which showed multiple nodular deposits on abdominal wall, bowel and omentum and peritoneal biopsy revealed granulomatous inflammation with caseous type necrosis compatible with mycobacterium tuberculosis infection. This was confirmed by tuberculosis genome identification on the biopsy sample confirming a diagnosis of peritoneal tuberculosis with secondary portal vein thrombosis and cavernous formation due to local inflammation. The patient was started on anti-tuberculosis treatment and warfarin was discontinued, following which she made a remarkable recovery.

**Conclusion:**

Peritoneal tuberculosis can present with unusual manifestations such as portal vein thrombosis and transudative ascites causing a diagnostic dilemma. Ascitic fluid analysis is generally not diagnostic. Under such circumstances peritoneal biopsy should be performed as it has a good diagnostic yield and accuracy.

## Background

Peritoneal tuberculosis is an important problem in regions of the world where tuberculosis is still prevalent [1]. It can present with a spectrum of clinical manifestations ranging from ascites, its typical form, to unusual presentations like portal vein thrombosis. Atypical presentations can mislead clinicians and result in delayed diagnosis or misdiagnosis [2–5]. A high index of suspicion is required for diagnosis as analysis of peritoneal fluid for tuberculosis bacillus has not only proven to be ineffective, but also it may delay diagnosis, resulting in increased mortality [6]. In light of new evidence peritoneal biopsy through laparoscopy or laparotomy has emerged as the gold standard for diagnosis [6–8].

## Case presentation

**Fig 1:**
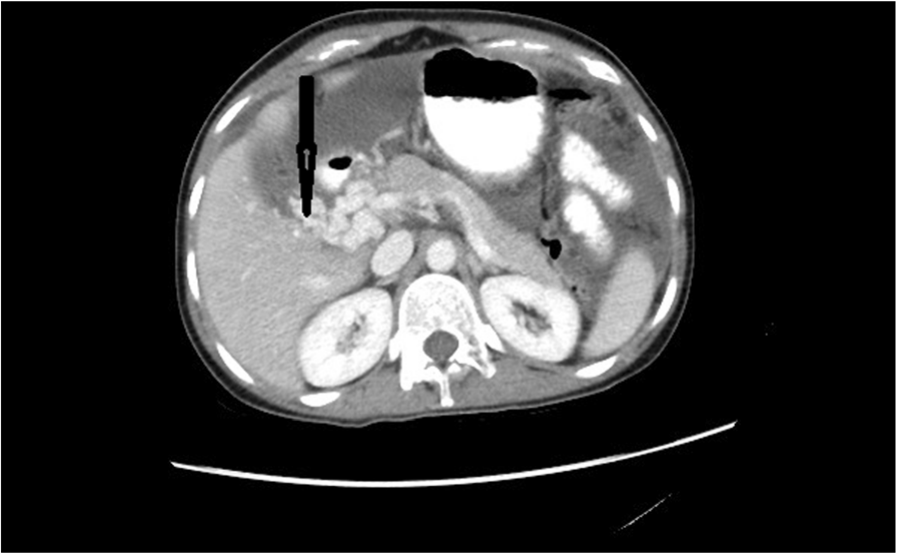
Thrombosed portal vein with cavernous transformation and dilated collaterals at the portal hilum (*Arrow*)

A 35 year old Sri Lankan house maid working in a Middle Eastern country had initially been investigated for progressive abdominal distension and diagnosed to have ascites with chronic portal vein thrombosis. Treatment with warfarin had been commenced suspecting an underlying thrombophilia but despite the treatment the abdominal distension worsened and she developed marked loss of appetite and loss of weight. Therefore, she decided to return to Sri Lanka for further investigation. There was no significant family history of thrombophilic conditions, personal history of thrombosis elsewhere, symptoms suggestive of systemic lupus erythematosus, the antiphospholipid syndrome or past history of intra-abdominal sepsis. She had no personal history or contact history of tuberculosis or symptoms of active pulmonary tuberculosis. Colonic, breast or ovarian malignancies were not documented among family members. On examination she was emaciated with a body mass index (BMI) of 18 kg/m^2^. There was no pallor, icterus, lymphadenopathy, photosensitive skin rashes, alopecia or oral ulcers. Peripheral stigmata of chronic liver cell disease were absent and facial or lower limb edema was not present. The abdomen was grossly distended with ascites. There was no hepatosplenomegaly or other abdominal or pelvic masses. Examination of the cardiovascular, respiratory and central nervous systems was unremarkable. Investigations revealed a normal full blood count {WBC 9.18 10^3^U/L (N 75, L 12, M 9.6, E 2.5 B O.2 %), Hb – 12 10^3^U/L, Plt 349 10^3^ U/L} with raised Inflammatory markers; CRP −139 mg/l, ESR – 70 mm/1 ^st^ hour. Liver function tests including serum albumin and coagulation profile were normal. Thrombophilic, antiphospholipid and autoimmune screenings were unremarkable. Chest X ray was normal. Peritoneal fluid analysis revealed a transudative ascites with lymphocytosis (WBC 1300/CMM, RBC 1500/CMM, Polymorphs 8%, and Lymphocytes 92 %) Serum to ascites albumin gradient (SAAG) was 1.3 g/dl. Contrast Enhanced Computed Tomography of the abdomen and pelvis revealed moderate ascites and chronic portal vein thrombosis with cavernous formation (Fig. 1), but no evidence of portal hypertension. There were no abdominal or pelvic masses. Mantoux test was positive (20 mm) but gold quantiferon assay was negative. Ascitic fluid adenosine deaminase level (ADA) was in the non-tuberculosis range and peritoneal fluid for acid fast bacilli staining and PCR for tuberculosis genome detection were negative. Peritoneal fluid culture revealed no growth. Thrombophilic conditions, intra-abdominal malignancy and sepsis having been ruled out we were left in a diagnostic dilemma. There was no evidence of tuberculous peritonitis apart from the high index of suspicion due to high background prevalence. So, three weeks after the peritoneal fluid analysis we managed to perform a laparoscopy which revealed multiple nodular deposits in the abdominal wall, bowel and omentum.Peritoneal biopsy showed granulomatous inflammation with caseous type necrosis compatible with mycobacterial tuberculosis infection. Subsequently PCR identified tuberculosis genome on the biopsy sample. Ultimately a diagnosis of peritoneal tuberculosis complicated by chronic portal vein thrombosis was made. The patient was referred to the central anti-tuberculosis treatment unit and commenced on standard anti-tuberculosis treatment regimen with fixed drug combination of Isoniazid, Ethambutol, Rifampicin and Pyrazinamide. Subsequently warfarin was discontinued .On follow up, one month later her liver enzymes were noted to be elevated. Therefore, all drugs were withheld and introduced gradually at weekly intervals with close monitoring of the liver functions. Within one month liver enzymes returned to baseline and she was reestablished on the standard regimen. Treatment was continued according to the current guidelines for a total of seven months including the month during which the drugs were reintroduced. Ultrasound scan of the abdomen performed a few weeks after reestablishing standard treatment showed resolution of ascites. She made a remarkable recovery at the end of treatment with no further complications.

## Discussion

Peritoneal tuberculosis is an important health concern in parts of the world where its prevalence is still high. Peritoneum is an uncommon site of extra pulmonary infection and the risk is increased in patients with cirrhosis, HIV infection, diabetes mellitus, underlying malignancy, following treatment with anti-tumor necrosis factor (TNF) agents, and in patients undergoing continuous ambulatory peritoneal dialysis (CAPD) [1, 9]. Infection most commonly results from reactivation of latent tuberculous foci in the peritoneum that were established following hematogenous spread from a primary lung focus [1]. Less frequently the organisms can enter the peritoneal cavity transmurally from an infected small intestine or contiguously from tuberculous salpingitis [10]. Although the patient had no direct contact history or any of the listed predisposing factors, Sri Lanka is a country with high background prevalence, and she may have had primary subclinical infection with hematogenous spread and subsequent reactivation. According to the available literature the majority of patients present with ascites and constitutional symptoms such as anorexia, fever and loss of weight [2, 11, 12]. It is more prevalent in females and seen more commonly in the third and fourth decades of life [2, 12]. Portal vein thrombosis is a rare manifestation of the disease and has been described mainly in case reports [3–5]. A high index of suspicion is needed for diagnosis of peritoneal tuberculosis and it should be included in the differential diagnosis of unexplained lymphocytic ascites with SAAG < 1.1 g/dl [11] In contrast , though lymphocytic predominant, ascites was a transudate in this case even in the absence of concomitant portal hypertension or cirrhosis. This again is unusual but the study carried out by Manohar A et al. describes such presentations [2]. Examination of the ascitic fluid including staining for acid fast bacilli is known to have a very low yield and the mortality associated with waiting for culture results has been demonstrated to be high [6]. In contrast, peritoneal biopsy either by laparoscopy or laparotomy has been proven in several studies to be the gold standard [6–8]. In this patient all diagnostic modalities including tuberculosis genome detection tests on ascitic fluid were inconclusive making the diagnosis a dilemma. But high index of suspicion and peritoneal sampling through laparoscopy ultimately led to the correct diagnosis. In the majority the standard treatment as for pulmonary tuberculosis leads to rapid clinical improvement [13].

## Conclusion

Peritoneal tuberculosis can present with unusual manifestations like portal vein thrombosis and transudative ascites in the absence of portal hypertension making the diagnosis a dilemma. Ascitic fluid analysis is generally inconclusive. Under such circumstances peritoneal biopsy should be performed as it has a good diagnostic yield and accuracy.

## Consent statement

Written informed consent was obtained from the patient for publication of this case report and any accompanying images. A copy of the written consent is available for review by the Editor of this journal.

## Abbreviations

BMI: Body mass index
CRP: C reactive protein
ESR: Erythrocyte sedimentation rate
SAAG: Serum to ascites albumin gradient
CT: Computed tomography
ADA: Adenosine deaminase level
PCR: Polymerase chain reaction
TNF: Tumor necrosis factor
CAPD: Chronic ambulatory peritoneal dialysis
